# A Novel Aortic End-to-End Anastomotic Stapler Device—Results of a Human Cadaver Study

**DOI:** 10.1093/ejcts/ezag015

**Published:** 2026-01-22

**Authors:** Emilio Osorio-Jaramillo, Jasmine El-Nashar, Satoshi Kainuma, Ewald Unger, Wilhelm Schreiner, Mohamed El Din, Paata Pruidze, Giorgi Didava, Wolfgang J Weninger, Daniel Zimpfer, Marek P Ehrlich

**Affiliations:** Department of Cardiac and Thoracic Aortic Surgery, Medical University of Vienna, A-1090 Vienna, Austria; Division of Anatomy, Center for Anatomy and Cell Biology, Medical University of Vienna, A-1090 Vienna, Austria; Department of Cardiac and Thoracic Aortic Surgery, Medical University of Vienna, A-1090 Vienna, Austria; National Cerebral and Cardiovascular Center, 564-8565 Suita, Osaka, Japan; Center for Medical Physics and Biomedical Engineering, Medical University of Vienna, A-1090 Vienna, Austria; Center for Medical Physics and Biomedical Engineering, Medical University of Vienna, A-1090 Vienna, Austria; Division of Anatomy, Center for Anatomy and Cell Biology, Medical University of Vienna, A-1090 Vienna, Austria; Division of Anatomy, Center for Anatomy and Cell Biology, Medical University of Vienna, A-1090 Vienna, Austria; Division of Anatomy, Center for Anatomy and Cell Biology, Medical University of Vienna, A-1090 Vienna, Austria; Division of Anatomy, Center for Anatomy and Cell Biology, Medical University of Vienna, A-1090 Vienna, Austria; Department of Cardiac and Thoracic Aortic Surgery, Medical University of Vienna, A-1090 Vienna, Austria; Department of Cardiac and Thoracic Aortic Surgery, Medical University of Vienna, A-1090 Vienna, Austria

**Keywords:** aortic anastomotic device, end-to-end anastomosis, human cadaver model, aortic surgery, circulatory arrest

## Abstract

**Objectives:**

To evaluate a novel aortic anastomotic device designed for end-to-end anastomosis between a vascular prosthesis and the native aorta, aiming to facilitate aortic replacement and shorten circulatory arrest times.

**Methods:**

Ascending aortic replacements with Dacron grafts and external Teflon felt strips were performed in 10 fresh human cadavers. Five procedures were performed using a continuous 4-0 polypropylene running suture, and 5 with the aortic anastomotic device. The device deploys single straight needle pins through the aortic wall and felt, securing them externally with a cap. Explanted graft-aorta specimens were connected to a pressure pump filled with glycerol/H_2_O solution (170/90 mm Hg, 5 minutes). The primary end-point was anastomotic time; secondary end-points were fluid loss and tissue trauma assessed macroscopically and microscopically.

**Results:**

Median time for end-to-end anastomosis was significantly shorter with the aortic anastomotic device (05:39 [interquartile range, IQR 04:22-06:30] minutes:seconds) compared to conventional suture (09:17 [IQR 06:53-10:34]; *P *= .016). The number of pins per anastomosis was lower than the number of stitches (17 [15-18] vs 22 [21-24]; *P *= .008). Fluid loss was comparable between groups (100 [52.5-174.5] mL vs 45 [21.8-104.0] mL; *P *= .310). Microscopy revealed intimal tears in all sutured specimens but in none of the device group, *P *= .008.

**Conclusions:**

In this preliminary human cadaver study, the novel aortic anastomotic device proved to be technically feasible, significantly faster than conventional suturing, and produced less tissue trauma while maintaining comparable sealing. The device may shorten circulatory arrest times and improve anastomotic quality in aortic surgery. Further *in vivo* studies are warranted.

**Clinical trial registration:**

This was a prospective experimental cadaver study, not eligible for registration in a public registry for clinical trials.

## Graphical abstract

**Figure:**
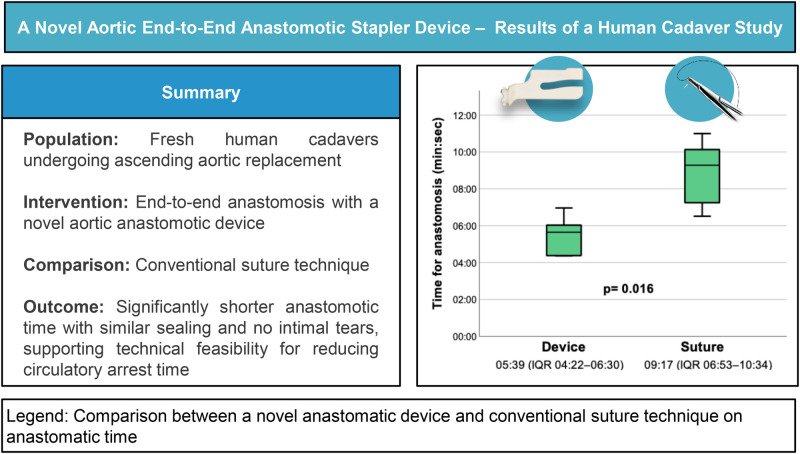

## Introduction

Aortic replacement with a vascular prosthesis remains the gold standard for the treatment of various aortic pathologies requiring surgical repair, such as ascending aortic aneurysms and type A dissections.^1^^,^^2^ One major challenge in these procedures is to perform a secure and durable distal anastomosis between the vascular graft and the native aorta.

In the present study, we evaluated a modified version of the aortic anastomotic device.^3^ This device uses a perpendicular pin-and-rivet fixation principle to join the vascular graft and native aorta. We tested the device in a standardized human cadaver model, directly comparing it with conventional suturing for distal anastomosis in ascending aortic replacement.

Conventionally the distal anastomosis is achieved using a continuous running suture, which can be time-consuming and technically demanding, particularly in patients requiring circulatory arrest. Prolonged circulatory arrest has been linked to increased risk of permanent and temporary neurological deficits, highlighting the clinical importance of strategies that shorten anastomotic time in order to improve outcomes.^4^^,^^5^

Previous attempts to develop mechanical or sutureless alternatives for aortic anastomoses^6–8^ have not resulted in devices that are currently applied in clinical practice. In a recent experimental study of acute type A dissection repair, we demonstrated feasibility^3^ when using the aortic anastomotic device for distal repair with the sandwich technique.^9^ Whether this concept can be reliably translated to direct end-to-end anastomosis between the aorta and a vascular prosthesis, however, has not yet been investigated.

Primary and secondary end-points of this study included anastomotic time, sealing properties and macro and microscopic assessment of tissue trauma.

## Methods

This was a prospective experimental study performed in a standardized human cadaver model. Following ethical approval, 10 randomly selected fresh human cadavers with a post-mortem time <72 hours and without prior aortic or cardiac operation were used.

The study was conducted in accordance with the principles of the Declaration of Helsinki. The Medical University of Vienna Ethics Review Board gave approval (on the October 25, 2024; No. EK 1997/2024). All individuals included in this study authorized the donation of their bodies through written informed consent during their lifetime.

**Figure 1.**
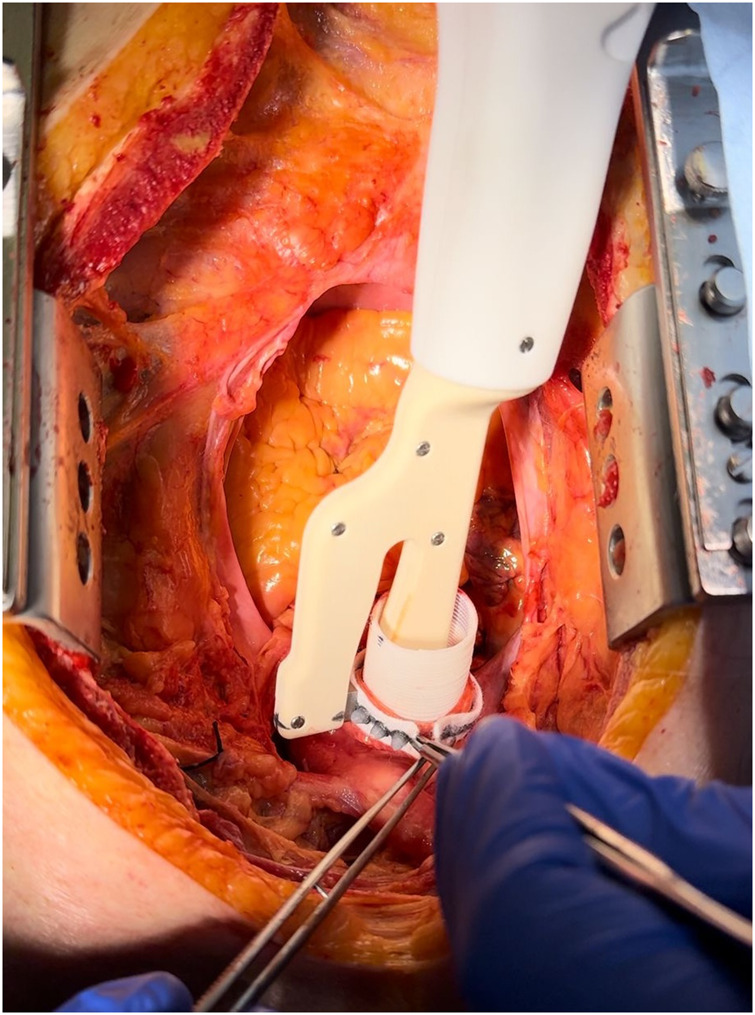
Intraoperative View of the Aortic Anastomotic Device Positioned Inside the Vascular Prosthesis During Implantation. Some pins have already been set, and the prosthesis is placed within the native ascending aorta for distal anastomosis.

**Figure 2.**
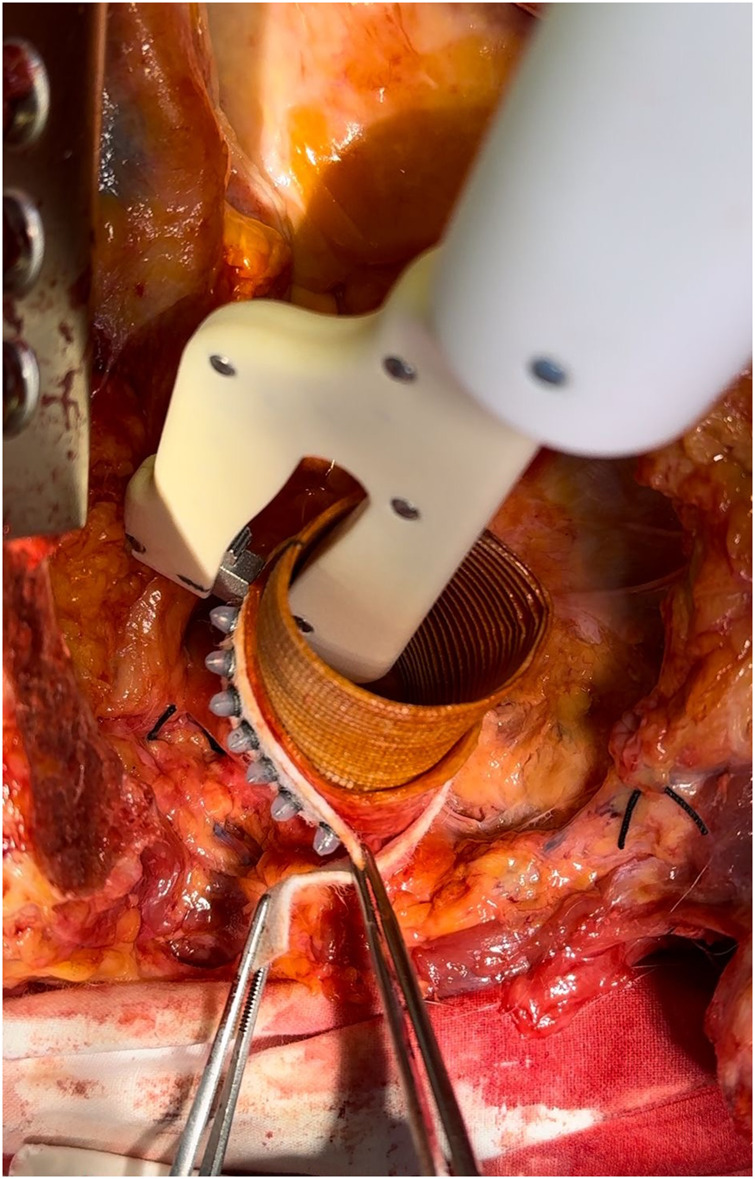
Intraoperative View at a Later Stage of the Procedure. From within the prosthesis, the device deploys straight needle pins through the prosthetic wall, the native aortic wall, and the circumferential felt strip, securing the graft to the aorta.

**Figure 3.**
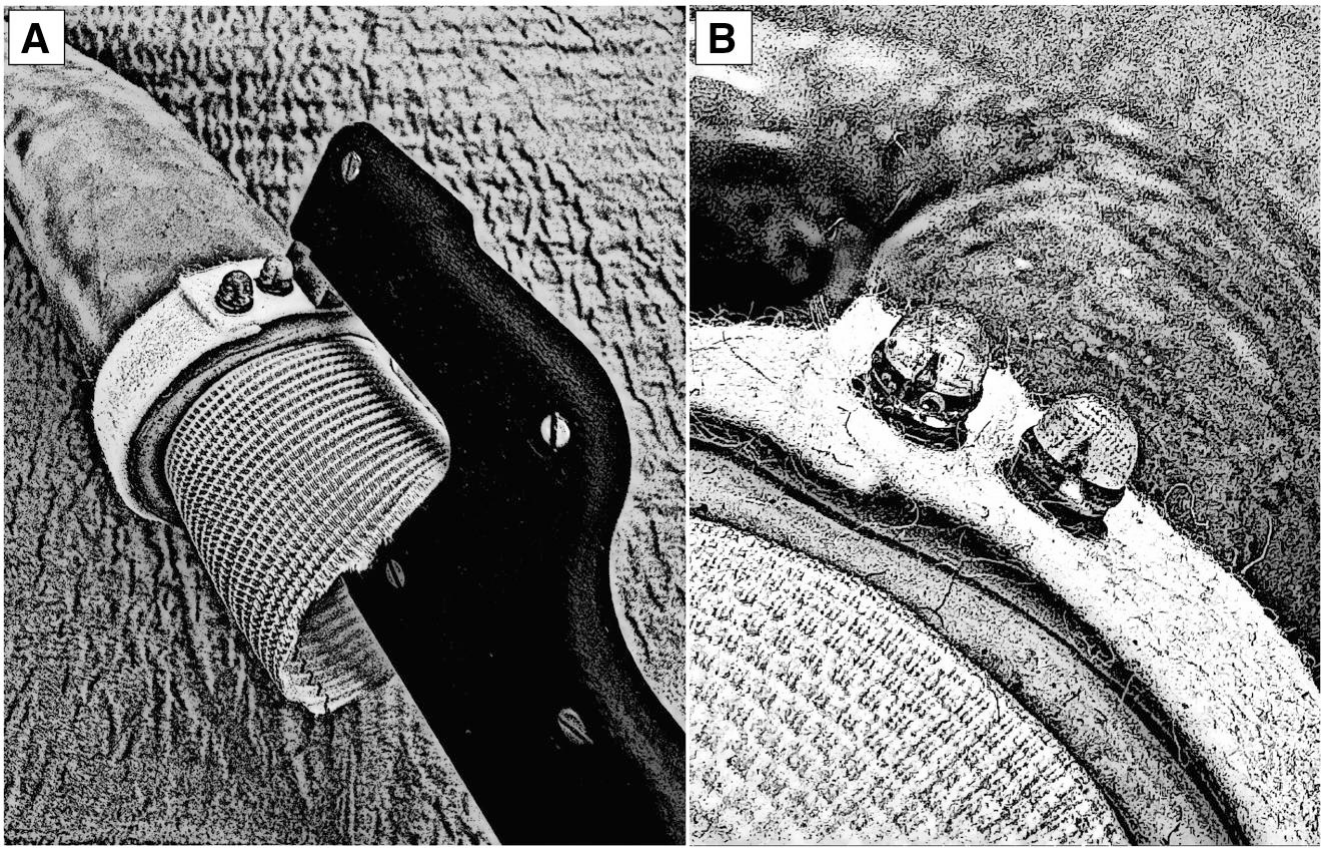
Illustration of the Aortic Anastomotic Device in Use. (A) The use of the device and the principle of the procedure. (B) Two deployed pins securing the prosthesis to the aortic wall and the circumferential felt strip. The needle tips are visible within the silicone caps.

Median sternotomy was performed, and the ascending aorta including the proximal aortic arch were exposed. The aortic anastomotic device was used for the distal anastomosis between a standard Dacron graft and the native ascending aorta (see **Figures 1 and 2**). The device consists of 2 handles and exchangeable single-use heads, each containing a rivet-like pin. Both the handle and the needle head are designed as single-use components, not intended for re-sterilization. For each pin placement, a new head is mounted on the handle and a straight titanium pin (diameter 0.6 mm, length 8.5 mm) is deployed from the luminal side outward through the Dacron graft, the aortic wall and an external felt strip, where it is secured externally by a silicon cap that provides compression of the graft-aorta-felt interface (illustrated in **Figure 3**). While one pin is placed, the scrub nurse prepares the next head, allowing a continuous workflow (see **Video 1**). In the control group, the anastomosis was performed using a conventional continuous 4-0 polypropylene running suture. In both groups, the suture line was externally reinforced with a circumferential Teflon felt strip. Time measurement started with the first manoeuvre (first stitch or device application) and ended with completion of the anastomosis. Time measurement included all steps of the procedure, including the exchange of needle heads between pin deployments, to accurately reflect clinical workflow. All anastomoses were performed by a single experienced cardiovascular surgeon without any financial or commercial involvement in the investigated device, in order to reduce operator-related variability.

Explanted graft-aorta specimens were connected to a closed-loop perfusion circuit filled with a methylene-blue-stained solution of 40% glycerol and 60% water, approximating blood viscosity.^10^ The circuit delivered a pulsatile flow (systolic phase 1s, diastolic phase 5s) at set pressures of 160/90 mm Hg. Leakage volume was collected and quantified over a period of 5 minutes.

Following perfusion, all anastomoses were fixed in 4% paraformaldehyde solution and were examined both macroscopically and microscopically. To assess the tissue’s integrity, suture and needle channels were analysed. In the suture group, all stitches were removed using fine microscissors under magnification and without traction on the aortic wall. This approach enabled the identification of whether a tear was present before or after removal. In addition, all observed intimal tears were located within stitch channels rather than along the suture line or in adjacent areas, suggesting that tissue damage resulted from needle penetration rather than from the removal process. Specific end-points included the presence of intimal tears and intimal flaps.

### Statistics

Data collection was performed by an investigator without any financial or commercial involvement in the device, in order to minimize potential bias. Statistical analyses were conducted independently by the institutional Department of Medical Statistics. Investigators with potential conflicts of interest had no access to the raw data prior to completion of the analysis. Continuous variables, including anastomotic time and leakage volume, are presented as median values with interquartile ranges (IQRs). Both groups (device and suture group) were compared, including the body donor’s characteristics and the prespecified outcome variables. The primary end-point was anastomotic time; secondary end-points were fluid loss and tissue trauma. Fisher’s exact test was used for categorical variables and the Mann-Whitney *U*-test for independent continuous variables. A 2-sample Kolmogorov-Smirnov test was additionally performed to compare the distributions of fluid loss between groups. This was a preclinical feasibility study with a small sample size, and all statistical comparisons were exploratory. Therefore, correction for multiple comparisons was not performed and *P*-values should be interpreted as descriptive rather than confirmatory. Two-sided *P*-values <.05 were defined as statistically significant. SPSS 29.0.0 (IBM Corp., Armonk, NY, United States) was used for statistical analyses.

## Results

Ten cadavers with a median age of 83 years (IQR 79-86) were included. Median aortic diameter was 29 mm (IQR 26-32), and median wall thickness 1.4 mm (IQR 1.3-1.8).

Baseline characteristics did not differ between the device and the suture groups (see **Table 1**).

**Table 1.** Baseline Characteristics of Cadavers included in the Study

Variable	Overall (*n* = 10)	Device (*n* = 5)	Suture (*n* = 5)	*P*-value
Age (years)	83 (81-86)	83 (79-86)	83 (80-85)	1.000
Sex, female, no. (%)	4 (40)	2 (40)	2 (40)	1.000
Aortic diameter (mm)	29 (26-32)	28 (27-33)	30 (25.5-32)	1.000
Aortic wall thickness (mm)	1.40 (1.30-1.83)	1.40 (1.30-1.70)	1.30 (1.30-1.90)	1.000
Prosthesis diameter (mm)	26 (23.5-28.0)	26 (23-29)	26 (23-28)	1.000

Values are presented as median (interquartile range, IQR) unless otherwise indicated.

Median prosthesis diameter was 26 mm (IQR 23.5-28) with no difference between groups (device: 26 mm [IQR 23-29] vs suture: 26 mm [IQR 23-28]; *P *= 1.000).

Procedural and outcome data are summarized in **Table 2**.

**Table 2.** Procedural and Outcome Parameters Comparing the Aortic Anastomotic Device With Conventional Suturing

Variable	Overall (*n* = 10)	Device (*n* = 5)	Suture (*n* = 5)	*P*-value
No. of sutures/pins per anastomosis	20 (17-22)	17 (15-18)	22 (21-24)	.008
Time for end-to-anastomosis (minutes:seconds)	06:44 (05:20-09:29)	05:39 (04:22-06:30)	09:17 (06:53-10:34)	.016
Fluid loss (mL)	73.50 (23.88-152.50)	100 (52.50-174.50)	45 (21.75-104.00)	.329
Presence of intimal tear, no. (%)	5 (50)	0 (0)	5 (100)	.008
No. of microscopic intima flaps	1 (0-2)	0 (0-0)	2 (2-4)	.008

Values are presented as median (interquartile range, IQR) unless otherwise indicated. *P*-values are based on Mann-Whitney *U*-test for continuous variables and Fisher’s exact test for categorical variables. A 2-sample Kolmogorov-Smirnov test was performed to compare distributions of fluid loss.

The number of fixation points was lower with the device (17 [IQR 15-18]) compared with the number of stitches in the suture group (22 [IQR 21-24]; *P *= .008). In total, 83 pins were successfully placed.

### Primary end-point

Median anastomotic time was significantly shorter with the device compared to suturing (05:39 minutes [IQR 04:22-06:30] vs 09:17 minutes [IQR 06:53-10:34]; *P *= .016) (see **Table 2**).

### Secondary end-points and microscopy

Leakage volumes were comparable between groups (device: 100 mL [IQR 52.5-174.5] vs suture: 45 mL [IQR 21.8-104.0]). A 2-sample Kolmogorov-Smirnov test demonstrated no difference in the distributions of fluid loss between groups (*P *= .329).

**Figure 4.**
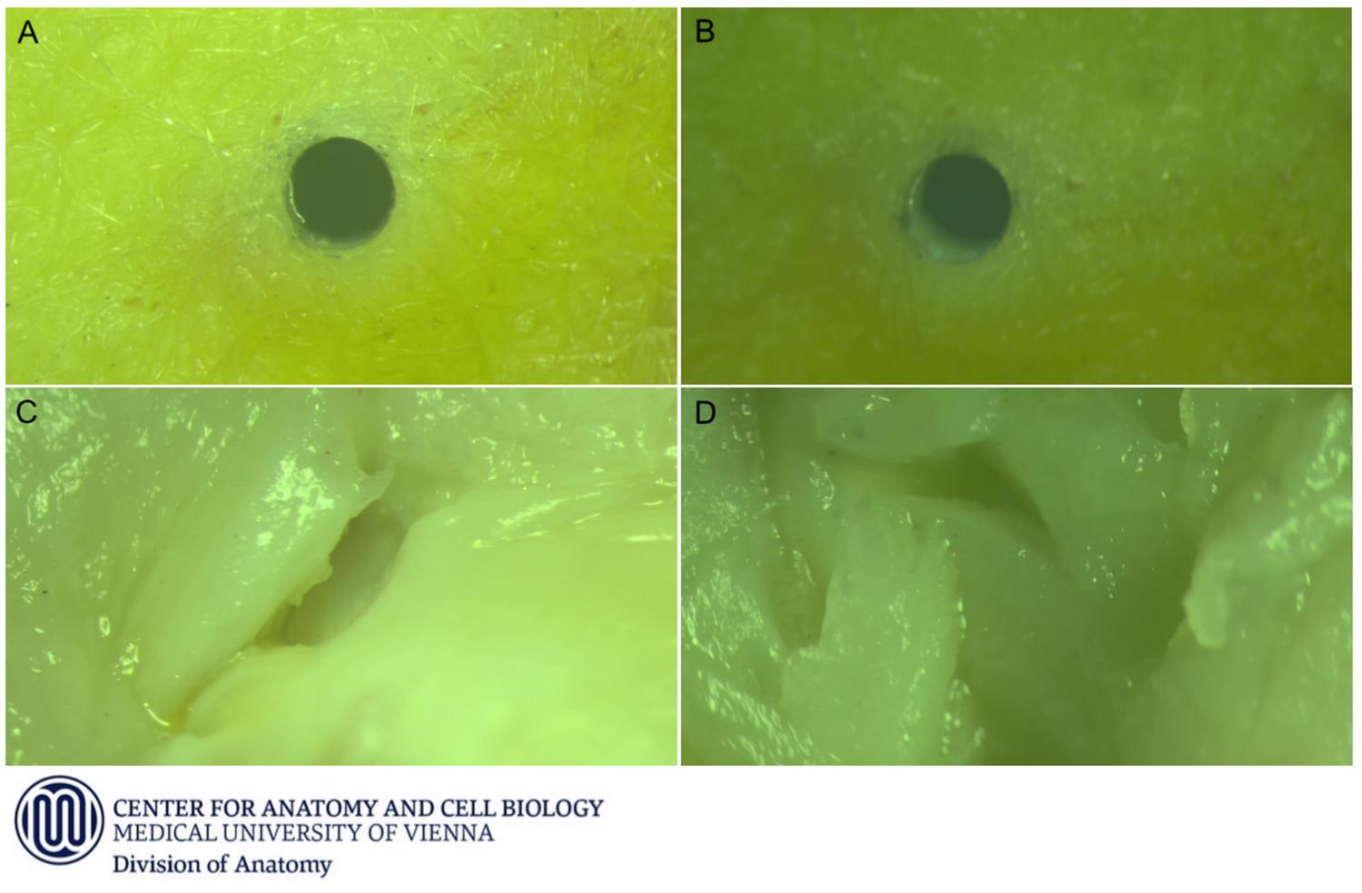
Representative Microscopic Findings. (A and B) Two pin sites after removal of the rivets in the device group. (C and D) Intimal tears within stitch channels observed in the suture group.

Histological examination revealed intimal tears in all sutured specimens but in none of the device anastomoses (*P *= .008). The number of microscopic intima flaps per individual was higher in the suture group (2 [IQR 2-4]) than in the device group (0 [IQR 0-0]; *P *= .008) (see **Table 2**). Examples from the microscopic findings of the device group and of the suture group are given in **Figure 4**.

In exploratory analyses, no correlations were observed between aortic diameter, age, or wall thickness and any of the procedural outcomes, including anastomotic time, leakage volume, or microscopic tissue trauma.

## Discussion

In this preclinical cadaveric study, we evaluated a novel aortic anastomotic device for direct end-to-end anastomosis between a vascular prosthesis and the native aorta. The main findings were that the device enabled significantly shorter anastomotic times compared with conventional suturing,^1^^,^^2^ required fewer fixation points, and resulted in less tissue trauma, while providing comparable sealing properties.

Performing a secure and durable distal anastomosis is a critical step in ascending aortic replacement.^1^^,^^2^ Conventionally, this is achieved using a continuous running suture,^1^^,^^2^ which is reliable but time-consuming and technically demanding. The reduction in anastomotic time achieved with the device in this study is clinically relevant, as shortening circulatory arrest duration is a major determinant of postoperative neurological risk and overall outcomes in complex aortic surgery.^4^^,^^5^^,^^11^^,^^12^

Importantly, microscopic analysis demonstrated less tissue trauma with the device compared to suturing, suggesting that automated pin placement from inside to outside may reduce intimal tearing and flap formation. This feature may be particularly advantageous in fragile aortic tissue, such as in patients with aneurysmal disease,^13–15^ where minimizing manipulation of the vessel wall is crucial. Additionally, potential clinical implications may include a reduced risk of embolic complications or future pseudoaneurysm formation when intimal trauma is minimized. Comparable leakage rates between groups further indicate that the sealing properties of the device are not compromised. However, it is important to mention that leakage volumes were numerically higher in the device group and due to the small sample size, a type II error cannot be excluded. Nevertheless, the absolute values remained within an acceptable range observed in non-coagulable cadaver models,^3^^,^^10^^,^^16^ and *in vivo* leakage may therefore be overestimated. In a clinical setting, acceptable blood loss during aortic anastomosis is strongly influenced by coagulation, local tamponade, perioperative haemostatic management, as well as native aortic wall tension and elasticity, none of which are present in the applied model. All in all, the observed numerical trend towards higher leakage should be interpreted with caution—it does not necessarily indicate inferior sealing performance *in vivo*. Lastly, future studies in perfused large-animal models will be necessary, especially to evaluate haemostatic performance, device handling, visibility in limited operative space, and anastomotic integrity under physiological coagulation and blood pressure, as well as performance under realistic conditions on cardiopulmonary bypass, including hypothermia and systemic anticoagulation. These conditions cannot be assessed in the present cadaver model. Furthermore, no long-term data on durability, healing response, infection risk, or biological compatibility can be derived from this study.

In addition to procedural benefits, the device introduces a fundamentally different fixation concept compared with earlier automated or sutureless systems. Whereas previous approaches often relied on lateral stapling or clip compression, the present design applies a perpendicular pin-and-cap mechanism that achieves a rivet-like fixation while minimizing shear traction on the vessel wall. This may explain the absence of intimal tears in the device group and represents a notable design improvement over earlier systems.

Previous attempts to develop mechanical or sutureless techniques for aortic anastomosis, particularly stapler-based systems, have not resulted in devices suitable for clinical use,^6–8^^,^^17^ likely for multiple reasons. One major limitation of earlier devices might have been their fixation principle: many relied on staples that compressed or deformed the vessel wall, potentially causing intimal damage or incomplete sealing. By contrast, the present device deploys straight needle pins that penetrate the aortic wall perpendicularly from the luminal side and are secured externally. Such a design could provide an important advantage in aneurysmal aortas, where tissue fragility often complicates conventional suturing or stapling.^15^ We also observed that fewer penetration sites were required than with conventional suturing, which may further reduce cumulative tissue trauma along the anastomotic line—an advantage that could be relevant in fragile or thinned aortas, although this must be validated *in vivo*.

Although large aneurysmal aortas were not included in this study, the device group contained segments with approximately 1 mm wall thickness and one specimen measuring 36 mm in diameter, without compromise of sealing or increased intimal trauma. These thinner samples provide preliminary support that perpendicular pin fixation may remain effective in reduced wall thickness. Because fixation forces are distributed through both an inner plate and an external cap that compress the graft-aorta-felt interface, rather than applied tangentially as with sutures, this principle may offer mechanical advantages in fragile or thinned aortic tissue. Nevertheless, validation in severely thinned and aneurysmal aortas is still required in future studies.

The learning curve for the device appears short, as its handling is intuitive and each pin is deployed through a simple trigger mechanism. During the procedure, the graft and external felt are maintained in position while the surgeon activates the handle, after which the next pre-loaded head is immediately provided to enable a continuous workflow. Only minimal training is required, and dry practice on 2-3 silicone tubes appears sufficient to ensure consistent pin spacing and to avoid gaps.

In a recent cadaveric study from our group, we demonstrated the feasibility of the device in a model of acute type A dissection repair using the sandwich technique.^3^^,^^9^ The present study extends these findings by demonstrating reliable performance of the device for direct end-to-end anastomosis in intact aortas. Taken together, these results suggest that the device combines procedural speed with less traumatic fixation and may overcome key limitations that prevented earlier automated systems from clinical translation.

### Limitations

Several limitations must be acknowledged. First, this was a small preclinical study in human cadavers, which cannot fully replicate the physiological conditions of living patients, particularly with respect to tissue quality, coagulation, and haemodynamics. Second, outcome parameters were limited to procedural performance, leakage under standardized conditions, and microscopic analysis. Clinical end-points such as neurological outcomes or long-term durability cannot be assessed in this setting. Although the device is handheld, compact, and operates with a simple one-step trigger deployment, its performance in deep or restricted operative fields, like the distal aortic arch during hypothermic circulatory arrest, was not assessed in this study. These aspects, together with considerations of device complexity and cost, require evaluation in dedicated large-animal models incorporating realistic operative constraints and cardiopulmonary bypass conditions.

Given the small sample size, the study was not powered to detect associations between device performance and anatomical variables, like aortic diameter, age, or wall thickness. However, no apparent trends were observed in exploratory analyses. Finally, while the sample size was small and the results must therefore be interpreted as exploratory, even this limited number of experiments was sufficient to demonstrate the potential benefits of the device and to provide proof-of-concept.

## Conclusion

In conclusion, this cadaveric feasibility study demonstrates that the novel aortic anastomotic device enables significantly faster anastomosis compared with conventional suturing, which is particularly relevant in procedures requiring circulatory arrest. Sealing capacity was comparable between groups, as reflected by similar fluid loss. Microscopic analysis showed reduced tissue trauma with the device, a feature of particular importance in fragile aneurysmal aortic tissue. Taken together, these preliminary data support the technical feasibility of this approach and justify further evaluation in animal models and, ultimately, in clinical studies to confirm its potential benefits in aortic surgery.

## Data Availability

The data underlying this article will be shared on reasonable request to the corresponding author.
